# Impact of Gd, Pr, Yb, and Nd doping on the magnetic properties of Mg-ferrite nanoparticles

**DOI:** 10.1007/s10856-025-06859-6

**Published:** 2025-01-30

**Authors:** H. Aglan, I. A. Ali, B. M. Ali, S. A. Kandil

**Affiliations:** 1 Cyclotron Facility, Nuclear Research Center, Egyptian Atomic Energy Authority, Cairo, Egypt; 2 Labeled Compounds Department, Hot Labs. Center, Egyptian Atomic Energy Authority, Cairo, Egypt; 3 Nuclear Physics Department, Nuclear Research Center, Atomic Energy Authority, Cairo, Egypt

## Abstract

This study aimed to synthesize MgFe_1.9_Ln_0.1_O_4_ (where, Ln = Yb, Pr, Gd, and Nd) ferrite nanoparticles via the sol-gel process and investigate their structural, morphological, and magnetic properties for potential hyperthermia applications. X-ray diffraction analysis (XRD) confirmed the cubic spinel structure for all samples. Transmission electron microscopy (TEM) images revealed nanometer-scale dimensions and nearly spherical morphology. Vibrating sample magnetometer measurements (VSM) indicated superparamagnetic behavior, with decreasing saturation magnetization (Ms) observed as Ln^3+^ content decreased. Specific absorption rate (SAR) analysis at 198 kHz demonstrated the influence of Ln^3+^ substitution on magnetic properties. Compared to existing studies, Ln^3+^ substituted (Yb, Pr, Gd, and Nd) nanoparticles demonstrate tunable magnetic properties and enhanced SAR performance, offering a more efficient design for hyperthermia treatment of solid tumors.

**Graphical Abstract:**

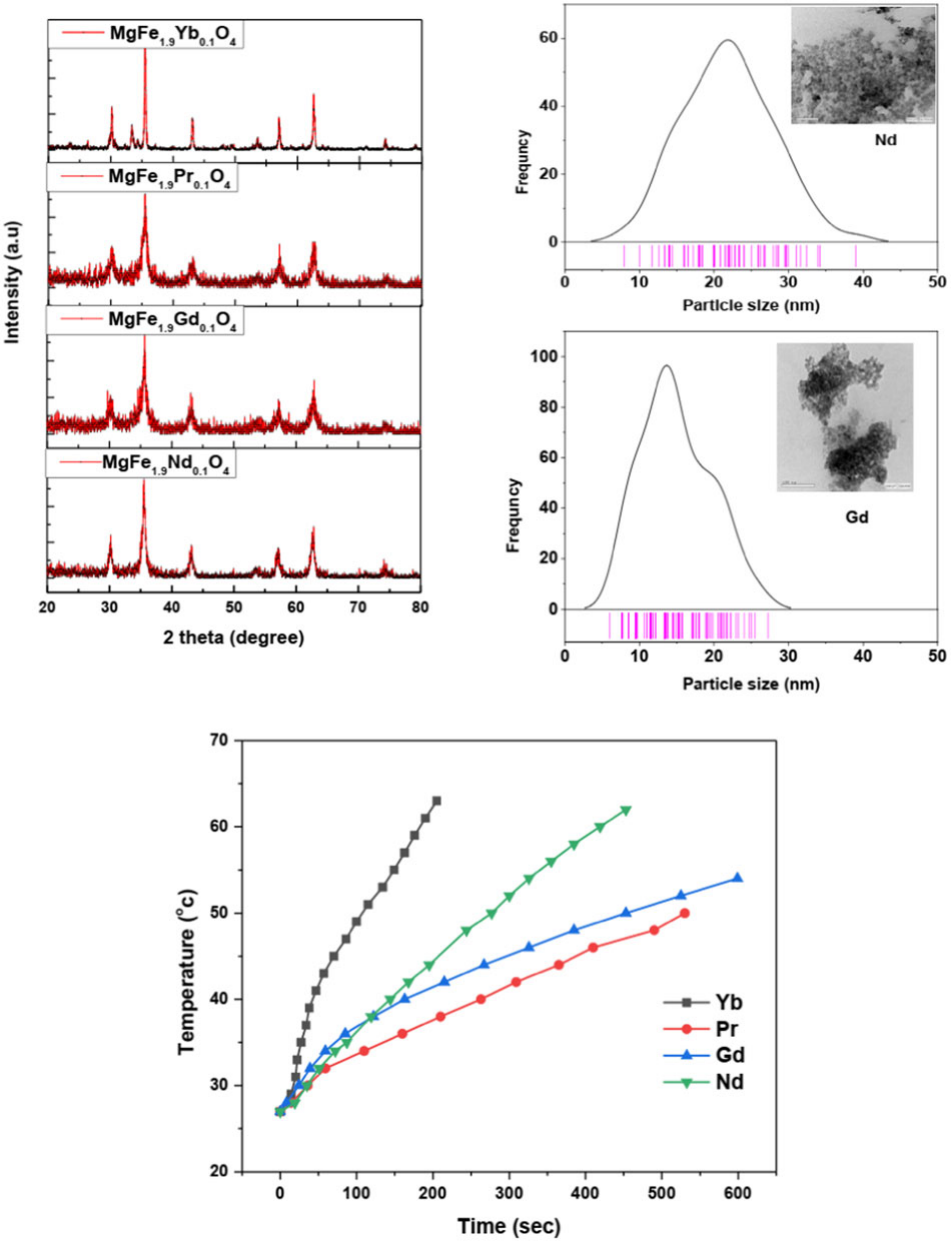

## Highlights

- Novel nanoferrite development: MgFe_1.9_Ln_0.1_O_4_ (Ln = Yb, Pr, Gd, Nd) nanoparticles were successfully synthesized via a simple sol-gel process.
- Tuneable magnetic properties: Ln^3+^ substitution effectively modulates saturation magnetization (M_s_) of the nanoparticles, highlighting a crucial factor for optimizing magnetic hyperthermia performance.
- Promising for hyperthermia: The study demonstrates a clear influence of Ln^3+^ content on the magnetic properties relevant for magnetic hyperthermia applications, suggesting potential for further development in cancer treatment.

## Introduction

Spinel ferrites (MFe_2_O_4_) exhibit fascinating structural, electrical, and magnetic properties. These properties are highly dependent on the type of cations present and their distribution within the crystal lattice (tetrahedral and octahedral sites). Preparation methods and conditions also significantly impact these characteristics. Due to their diverse functionalities, spinel ferrites have attracted considerable research interest for decades. Notably, their properties at the nanoscale differ markedly from their bulk counterparts. Studying nanoscale spinel ferrites is crucial due to their unique magnetic and electrical properties, coupled with exceptional chemical and thermal stability [1].

Magnesium ferrite (MgFe_2_O_4_) is a prominent magnetic oxide possessing a typical spinel cubic structure. However, its cation distribution is highly sensitive to heat treatment because of the high diffusibility of Mg^2+^ ions [2, 3]. Magnesium ferrites have found applications in microwave devices, magnetic recording media, transformer cores, noise filters, rod antennas, and even hold promise in heterogeneous catalysis, adsorption, sensors, and cancer treatment [4–9].

Rare earth elements boast excellent electrical insulation properties (high resistivity). Introducing them into spinel ferrites can potentially alter both electrical and magnetic characteristics. Additionally, these elements significantly influence the system’s magnetic anisotropy, making rare earth-substituted spinel ferrites promising alternatives to hexaferrites or garnets [10–12].

Partial substitution of Fe^3+^ ions with rare earth elements in the spinel structure leads to structural distortion and strain, significantly modifying magnetic and electrical properties [13–15]. Generally, due to their larger ionic radii, rare earth ions predominantly occupy the octahedral B-sites with limited solubility within the spinel lattice. However, previous studies have shown variations in the behavior of different rare earth elements within various spinel ferrites, leading to diverse structural properties [16].

Several methods exist for synthesizing soft spinel ferrite materials, including chemical co-precipitation, hydrothermal synthesis, mechano-chemical processing, microemulsion techniques, rheological phase reaction methods, and the sol-gel method [17–21]. Among these, the sol-gel process has gained significant attraction due to its applicability to a wide range of materials and its ability to control particle size, shape, and distribution [22].

Yttrium-doped iron oxide nanoparticles have been explored for magnetic hyperthermia. Doping with yttrium ions increased the magnetization of Fe_3_O_4_, enhancing its heating efficiency during hyperthermia treatment [23].

silica-encapsulated gadolinium-doped lanthanum strontium manganite nanoparticles were synthesized using the citrate–gel auto-combustion method. increasing gadolinium doping resulting in a reduction of the curie temperature of the nanoparticles. The specific absorption rate (SAR), which indicates the efficiency of heat generation, was also enhanced, suggesting improved therapeutic potential [24].

Magnetic properties of ferrite nanoparticles doped with various lanthanides, including gadolinium, dysprosium, and lutetium were studied. The results showed that saturation magnetization increased with lanthanide substitution. These superparamagnetic behaviors are advantageous for magnetic hyperthermia, as they facilitate efficient heat generation under an alternating magnetic field [25].

Eu-doped Fe_3_O_4_ nanoparticles functionalized with chitosan and dextran were also designed, representing how biopolymer encapsulation offers a promising approach to control agglomeration of Fe_3_O_4_ nanoparticles [26].

While extensive research has explored magnesium-based ferrites for diverse applications [27–29], reports investigating the impact of rare earth substitution on the magnetic properties, induction heating properties [30] of the Mg-ferrite system remain scarce.

This work aims to investigate the effect of rare earth ion substitution and distribution on the structural, magnetic, and morphological properties of MgFe_1.9_Ln_0.1_O_4_ (Ln = Yb, Pr, Gd, and Nd) ferrite nanoparticles synthesized using the sol-gel auto-combustion method.

## Experimental technique

This study employed the sol-gel method to synthesize Mg-ferrite nanoparticles with the formula MgFe_1.9_Ln_0.1_O_4_ (Ln = Yb, Pr, Gd, and Nd). Analytical grade reagents, Mg(NO_3_)_2_.6H_2_O, Fe(NO_3_)_3_.3H_2_O, Yb(NO_3_)_3_, Pr(NO_3_)_3_, Gd(NO_3_)_3_.6H_2_O, Nd(NO_3_)_3_.6H_2_O, and monohydrate citric acid (C_6_H_10_O_8_) from Sigma Aldrich, were used without further purification.

A stoichiometric solution of metal nitrates was prepared by dissolving appropriate quantities of metal nitrates and citric acid in double-distilled water, maintaining a 1:1 molar ratio. The mixture was continuously stirred at 300 rpm using a magnetic stirrer. The pH of the solution was found to be 3. Subsequently, the solution was heated at 90 °C for 30 min, leading to the formation of a viscous gel. The gel was subsequently dried and calcined in a muffle furnace with a heating rate of 10 °C/min. The temperature was maintained at 300 °C for two hours, followed by cooling to room temperature at a controlled cooling rate of 10 °C/min, resulting in the formation of the final crisp powder. The final powder was grinded in agate mortar to complete the measurements.

### Characterization techniques

X-ray diffraction (XRD): an X-ray diffractometer with CuK_α_ radiation (λ = 1.5406 Å) was used to determine the lattice parameter, crystallite size, and XRD patterns of the investigated samples. The scanning range was 2θ from 5 to 80° with an X-ray power of 40 kV and a current of 40 mA.

Transmission electron microscopy (TEM): The morphology of the particles was examined using a Jeol JEM-2100 transmission electron microscope. For particle size measurements, at least 80 to 90% of the particles in each image were measured using ImageJ software. The reported values represent the mean of these measurements. The standard deviation was used to determine the error percentage, which did not exceed 5%.

Vibrating sample magnetometer (VSM): Magnetic properties (saturation magnetization, M_s_, and coercive field, H_c_) of the nanoparticles were measured at room temperature using a Lake Shore Model 7410 (USA) VSM with a maximum applied field of 31 kOe.

### Specific absorption rate (SAR) measurement

Sample preparation: a homogeneous suspension of the nanomagnetic ferrites was prepared for SAR measurements. A mass of 0.1 g of ferrite powder was mixed with a saturated sodium citrate solution to achieve good dispersion. The solution was then subjected to sonication in an ultrasonic bath for 60 min at 60 °C. The suspension was centrifuged for 15 min at 2500 RPM. The powder was rinsed with acetone followed by double-distilled water for further purification. Finally, the re-suspended solution was obtained by sonicating for 30 min at room temperature and diluting it in 4 mL of double-distilled water.

### SAR measurement setup

An induction heater (DW-UHF-10kW, China) operating at a fixed frequency of 198 kHz was used to generate a magnetic field (H) of 9.27 kAm^−1^. The temperature of the solution was monitored using an optical fiber probe (model FOBS-2) immersed in the solution and connected to a digital meter (model OMEGA-FOB101). A computer interface software was employed to record the temperature variations for all samples. For SAR measurements, the error margins in the measurements are influenced by several factors, including potential powder loss during the washing process in sample preparation, the accuracy of sample weight measurements, the calibration and precision of the thermometer, and the effectiveness of thermal insulation during the measurement process. Collectively, these factors contribute to an estimated overall error margin of approximately 10%.

However, as the study primarily adopts a comparative approach, any errors introduced would uniformly affect all samples under identical conditions. This ensures that the observed trends and differences between samples remain valid, as systematic errors do not preferentially impact one sample over another.

## Results and discussion

### X-ray diffraction analysis

**Fig. 1:**
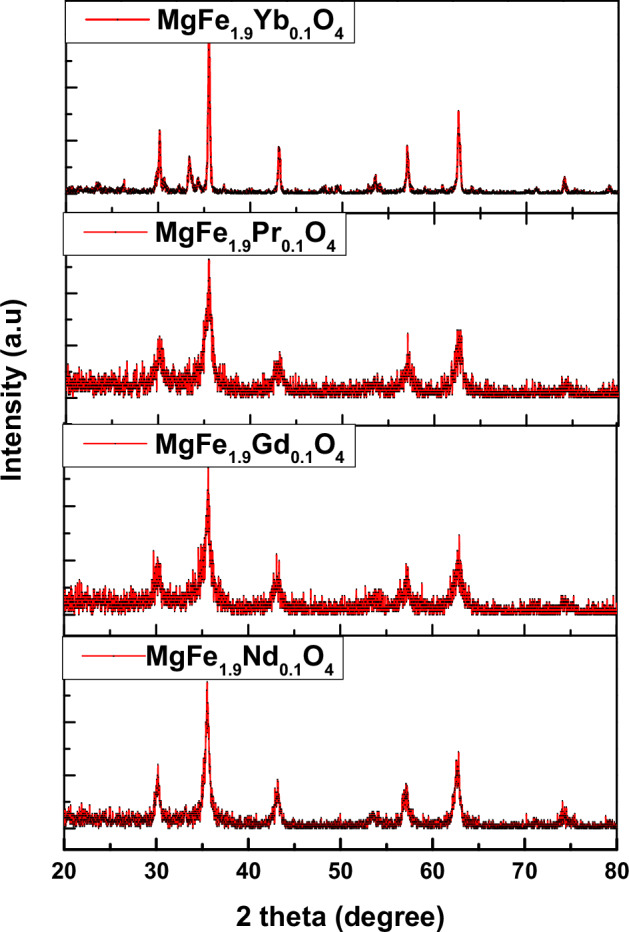
X-ray diffraction pattern and Rietveld refinement of MgFe_1.9_Ln_0.1_O_4_ where (Ln = Yb, Pr, Gd and Nd)

The XRD of MgFe_1.9_Ln_0.1_O_4_ where (Ln = Yb, Pr, Gd, and Nd) is shown in Fig. 1. The samples have a single spinel cubic crystal structure, and the diffraction peaks at 2θ = 30.17, 35.55, 43.21, 53.61, 57.05, and 62.65 correspond to reflections from the (220), (311), (400), (422), (511), and (440) crystal planes, respectively. The XRD patterns of the samples were compared with the Crystallography Open Database (COD) to confirm the phase. The broadening of the diffraction peaks demonstrates the nanoscale nature of the particles. The MgFe_1.9_Ln_0.1_O_4_ samples’ lattice parameter was investigated using Maud software and Rietveld refinement. The average crystallite size of MgFe_1.9_Ln_0.1_O_4_ samples is determined by Debye Scherrer formula as given in this equation [31],

1 $$\delta =\frac{0.92\lambda }{\beta \cos \theta }$$

where λ is CuK_α_ radiation, β is the full width at half maximum (FWHM) of the diffraction (311) peak and ‘θ’ is the diffraction angle. It is clear from Fig. 1 that the peak width of Yb-ferrite sample is the narrowest, which indicates to large particle size. The results of the Rietveld refinement analysis and crystallite size are tabulated in Table 1. It is obvious that lattice parameter of the investigated samples increased due to the generation of stresses and the expansions in the spinel lattice. This increase is due to the larger ionic radii of substituted metal ions than Fe^3+^ ions (0.64 Å) [32]. This increase is close to the ionic radii of the Ln ions, where the ionic radii of the Ln = Yb^3+^ [33], Nd^3+^ [34], Pr^3+^ [35] and Gd^3+^ [36] are 0.86, 0.98, 1.13 and 1.10 Å respectively. Moreover, it was found that the lattice parameter of the Ln samples is larger than that of Mg-ferrite (8.340 Å) [37]. This is due to the replacement of Fe^3+^ ions of small ionic radius by the Ln^3+^ ions of larger one. The strain (ε) induced in the crystal lattice because of doping in the investigated samples can be determined using the following equation [35]:

2 $$\varepsilon =\frac{{a}_{{doped}}-{a}_{{undoped}}}{{a}_{{undoped}}}$$

where *a*_undoped_ and *a*_doped_ is the lattice parameter of Mg-ferrite and doped samples, respectively. The calculated values of ε for each sample are listed in Table 1. The observed increase in strain can be attributed to the structural distortions induced in the crystal lattice of Mg-ferrite upon substitution of Fe^3+^ ions with larger lanthanide ions. It is noticed that ε increases as the ionic radius of the dopant increases. The larger ionic dopants disrupt the lattice uniformity, resulting in an elongation of the metal-oxygen bond lengths.

**Table 1:** The lattice parameter (*a*), crystallite size (δ), average particle size (D), Saturation magnetization (M_s_), Coercive field (H_c_), anisotropy constant (K) and SAR of the MgFe_1.9_Ln_0.1_O_4_ where (Ln = Yb, Pr, Gd and Nd) ferrites nanoparticles

	*Yb*	*Pr*	*Gd*	*Nd*
*a (A)*	8.350	8.378	8.365	8.358
*ε (×10*^*−3*^*)*	1.199	4.556	2.998	2.158
*δ (nm)*	35.06	11.45	8.03	12.26
*D (nm)*	36.2	11.9	9.5	22.0
*M*_*s*_*(emu/g)*	22.706	15.779	15.659	22.216
*H*_*c*_*(kOe)*	95.022	34.920	32.074	200.63
*K (J/m*^*3*^*)*	2247	574	523	4643
*SAR (W/g)* *±* *10%*	87.26	11.79	25.35	21.52

### TEM analysis

**Fig. 2:**
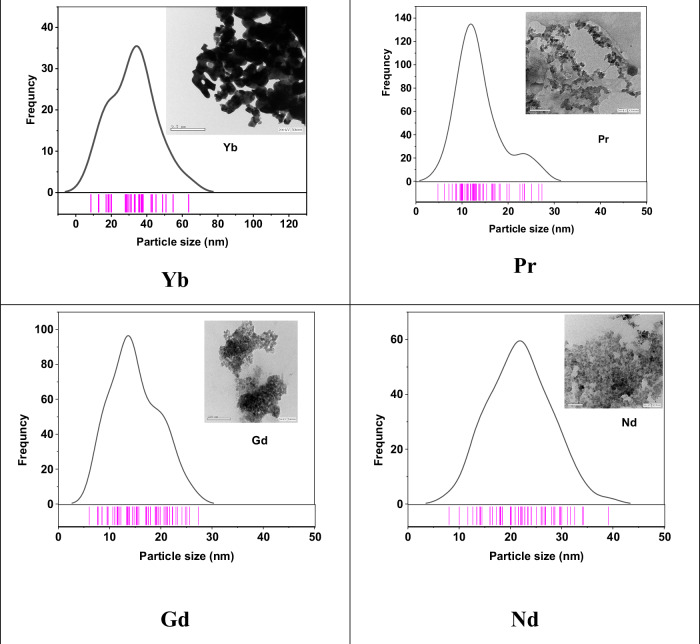
TEM images of MgFe_1.9_Ln_0.1_O_4_ nanomagnetic ferrite where (Ln = Yb, Pr, Gd and Nd)

Transmission electron microscopy (TEM) was employed to investigate the morphology and size distribution of the MgFe_1.9_Ln_0.1_O_4_ (Ln = Yb^3+^, Pr^3+^, Gd^3+^, and Nd^3+^) nanoparticles. Representative TEM images in Fig. 2 reveal semi-spherical nanoparticles with a homogeneous size distribution. ImageJ software (version 1.48 V) was employed to analyze the digital TEM images and determine the particle size distribution quantitatively. Table 1 summarizes the estimated particle size (D) values for each sample. The average particle size ranged from 9.5 to 36.2 nm, with the Yb-doped Mg-ferrites exhibiting the largest particles (36.2 nm) and the Gd-doped sample possessing the smallest (9.5 nm).

### Magnetic properties

**Fig. 3:**
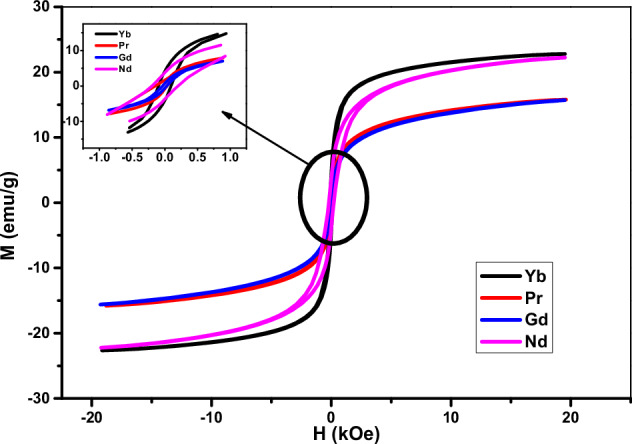
Magnetic hysteresis loops at room temperature for MgFe_1.9_Ln_0.1_O_4_ where (Ln = Yb, Pr, Gd and Nd)

Magnetic characterizations of MgFe_1.9_Ln_0.1_O_4_ were characterized by VSM at room temperature with the maximum applied field of 20 kOe. Typical magnetic hysteresis loops of the samples are shown in Fig. 3. The magnetic parameters: saturation magnetization (M_s_), coercivity (H_c_), initial permeability (µ_i_), and calculated anisotropic (K) constant are shown in Table 1.

**Fig. 4:**
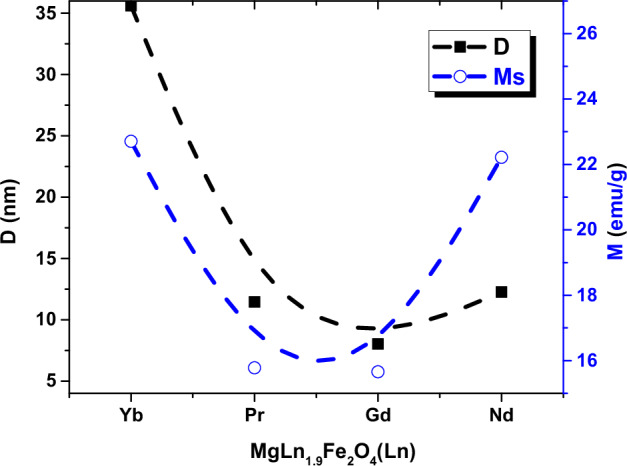
The relation between particle size (D) and saturation magnetization M_s_ vs. MgFe_1.9_Ln_0.1_O_4_ where (Ln = Yb, Pr, Gd and Nd) ferrites nanoparticles

The value of saturation magnetization depends on the grain size, preparation temperature, and died layer thickness [37]. The relation between saturation magnetization and particle size is shown in Fig. 4. With Ln^3+^ doping, the saturation magnetization value increases from 8 to 22.7 emu/g for MgFe_2_O_4_ [34] and Yb ferrite sample, respectively. This result can be attributed to the increase in the particle size (D) from 9.2 nm in the case of undoped Mg-ferrite to 36 nm in the case of doped. A decrease of the saturation magnetization can be described based on the cation dispersion and exchange interaction between iron (3d-orbitals) and between Ln^3+^ ions (4f-orbitals) at tetrahedral A and octahedral B sites. When Ln^3+^ ions are substituted at the expense of Fe^3+^ ions, some of the Fe^3+^ may migrate from B- to A-sites in view of the site preferences for different ions which leads to the increase of Fe^3+^ concentration at A-sites [38].

As shown in Table 1, the saturation magnetization (M_s_) increases with increasing particle size for all doped samples. This trend aligns with the stochastic model by Chakraverty and Bandyopadhyay [36], which describes the influence of particle size on magnetic properties in nanomagnets. According to this model, at room temperature and in the multi-domain regime, a larger surface-to-volume ratio in smaller particles leads to a higher surface spin disorder, resulting in lower M_s_. Conversely, larger particles exhibit a diminished surface contribution, enhancing the overall M_s_.

The variation of H_c_ with particle size (D) depends on the domain structure, which is influenced by critical size and surface interface anisotropy. In the single-domain region (particles below the critical size), H_c_ increases with increasing particle size, as described by Eq. (3)

3 $${H}_{c}=g-\frac{h}{{D}^{3/2}}$$

where g and h are constants. This is because smaller single-domain particles have lower energy barriers for domain wall movement, leading to lower coercivity.

Conversely, in the multi-domain region (particles above the critical size), H_c_ decreases with increasing particle size as expressed by:

4 $${H}_{c}=a+\frac{b}{D}$$

where a and b are constants. This can be attributed to the presence of multiple magnetic domains within larger particles, facilitating domain wall movement and reducing the overall coercivity.

Our experimental results align with these size-dependent trends, suggesting that the prepared nanoparticles exhibit a range of domain structures based on their particle sizes.

### SAR analysis

**Fig. 5:**
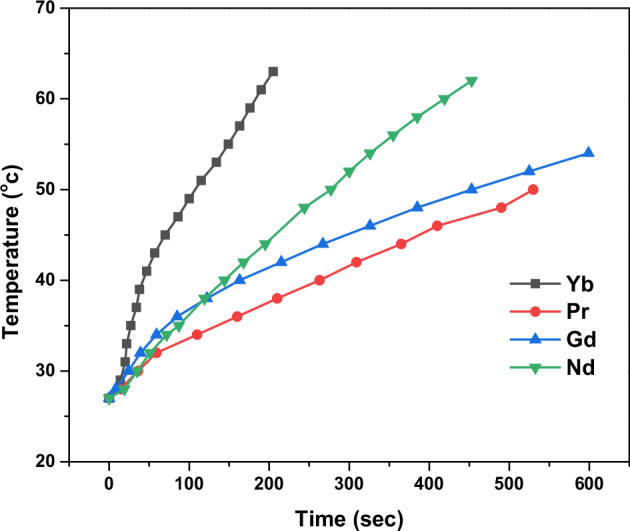
Temperature rate of MgFe_1.9_Ln_0.1_O_4_ where (Ln = Yb, Pr, Gd and Nd) at alternating magnetic fields

Figure 5 shows the temperature rise profiles of MgFe_1.9_Ln_0.1_O_4_ (where Ln = Yb, Pr, Gd, and Nd) with alternating magnetic fields. One can notice that the Yb-doped sample achieved the highest temperature rise, followed by the Nd-doped, Gd-doped, and Pr-doped samples. It is observed that the temperature linearly increases with time at the initial rise. The SAR values are calculated from this initial rise, adiabatic part, using Eq. [39].

5 $${SAR}=\frac{{C}_{W}{m}_{W}+{C}_{M}{m}_{M}}{{C}_{M}}\times \frac{\Delta T}{\Delta t}$$

where C_w_, C_M_ and m_W_, m_M_ are the specific heat and mass of water and magnetic particles, respectively. The SAR values are tabulated in Table 1. The sample with Ln = Yb recorded the highest SAR value while the sample with Ln = Pr is the lowest and the samples Ln = Gd and Nd are intermediate.

These results could be clarified as follows. It can be said that the SAR process depends on a complicated manner on several magnetic and structural parameters, which could combine to produce a heating rise in the samples. The SAR of the nanoparticles below the critical size is controlled by the two main loss: Neel τ_N_ and Brownian τ_B_ relaxation time. According to the following Eq. [36]

6 $${\tau }_{N}={\tau }_{0}{e}^{\frac{K{V}_{M}}{{kT}}}$$

7 $${\tau }_{B}=\frac{3\eta {V}_{H}}{{kT}}$$

The total time “τ” is

8 $$\tau =\frac{{\tau }_{B}{\tau }_{N}}{{\tau }_{B}+{\tau }_{N}}$$

where K is the anisotropy constant, V_M_ = 4πR^3^/3 the magnetic volume, R the radius of the particle, τ_o_ approximately = 10^−9 ^s, η the viscosity of the fluid, V_H_ called the hydrodynamic volume of the particle, which is larger than the magnetic volume V_M_, it is assumed that V_H_ = V_M_ × (1 + δ/R)^3^, where δ is the thickness of a surfactant layer. According to Eq. (6), the τ_N_ depends on the particle volume and anisotropy constant of the magnetic nanoparticles. In other words, the Neel loss exponentially rises as the particle volume and anisotropy decrease i.e. τ_N_ decreases. The Brownian process heat is produced by the friction between a rotating particle and the medium around it. Additionally, according to Brownian relaxation, the friction rises as the particle volume decreases, increasing the surface area i.e. τ_B_ decreases. On the other hand, over the critical size, the SAR process for the ferrimagnetic nanoparticles is directly proportional with the saturation magnetization.

In our case and as cleared in Table 1, the particle size for the samples with Ln = Pr and Gd is laying below the critical size ≤13 nm and the SAR values records 11.79 and 25.35 W/g, respectively. The observed decrease in the SAR value could be attributed to the increase in both D and K values, i.e., according to Eq. (6), with increasing D, the time required to perturbation the magnetic moment in the magnetic field increase which resulting the heating process to reduce. In addition, with increasing the anisotropy constant K, i.e. the energy required to return the magnetic moments to the set point, it causes delay in follow the applied field. Moreover, according to Eq. (7), the increase in V_H_ cause the Brownian process to decrease, which results in the SAR decrease [39].

In the region larger than the critical size, the dominant factor that affects the SAR process is the number of magnetic moments per unit volume (M_s_) beside the anisotropy constant in the crystal (H_c_). As seen in Table 1, in samples Ln = Yb and Nd the Ms is nearly equal while the Hc increase, which leads to a decrease in the SAR value. In another words, the magnetic moments in the crystal are unable to follow the applied magnetic field due to a rise in H_c_, which reduces the SAR value [39].

The SAR values of the investigated lanthanide-doped ferrite nanoparticles reached 87.26 W/g, which is approximately four times higher than that was reported for Mg-ferrite nanoparticles (19 W/g) [36]. This significant enhancement in the heating efficiency indicates the potential to reduce the required nanoparticle amount, in that way minimizing side effects and achieving therapeutic temperatures effectively.

## Conclusion

The XRD analysis confirms the formation of a single-phase spinel cubic structure for all of MgFe_1.9_Ln_0.1_O_4_ (Ln = Yb, Pr, Gd and Nd) nanoparticles. The lattice parameter increase, confirmed by Rietveld refinement, could be attributed to the replacement of larger Ln^3+^ ions (ionic radii: 0.86–1.13 Å) compared to Fe^3+^ (0.64 Å) into the spinel lattice, causing lattice expansion and generation of stress. These results demonstrate the successful synthesis of Ln-doped MgFe_2_O_4_ nanoparticles with tuneable particle size and tailored lattice parameters through rare earth element substitution.

The saturation magnetization (Ms) influence of surface spin disorder in larger particles. Ln^3+^ doping might lead to a decrease in Ms due to cation dispersion and the weakening of exchange interactions. The SAR values for the investigation samples correlate with their particle size and magnetic properties. Smaller particles (Pr and Gd) exhibit lower SAR compared to larger ones (Yb and Nd) likely due to a combination of factors. Additionally, the higher anisotropy constant (K) for smaller particles impedes their ability to follow the field’s direction. For larger particles (Yb and Nd), despite similar saturation magnetization (Ms), the higher coercivity (Hc) reduces their response to the field, leading to a decrease in SAR. Further investigations with a wider range of particle sizes and detailed analysis of relaxation mechanisms for each rare earth dopant are needed to fully understand the interplay between particle size, magnetic properties, and SAR in these materials. Optimizing particle size, coating, and surface functionalization of these materials with radioisotopes can significantly enhance their theranostic potential for applications in MRI/PET or MRI/SPECT modalities in the nuclear medicine field.
